# Modelling the behaviour of physiological processes: On the lack of a scientific basis in medical science

**DOI:** 10.1080/19420889.2025.2612465

**Published:** 2026-02-11

**Authors:** Abraham Peper

**Affiliations:** Department of Biomedical Engineering & Physics , Academic Medical Centre, University of Amsterdam, Amsterdam, The Netherlands

**Keywords:** physiological regulation, compensatory response, adaptation, homeostasis, feedback, drug dose, drug effect, homeopathy, mathematical model, drug tolerance, side effects

## Abstract

Physiological regulation is extremely complex and cannot be described by homeostasis, the mathematical model generally used in medical science. Homeostasis is based on assumptions which have never been tested or substantiated and when simulated appears not to be consistent with the real behavior of physiological regulation. As a consequence, drugs and drug treatments are developed on a trial and error basis, without a functional model guiding the process. This paper evaluates a mathematical model published previously which much better corresponds to the complex behavior of regulated physiological processes. The compensatory response – the reaction of the body’s defense mechanism to disturbances – is shown to be a major factor in the effects of drugs. Its magnitude at any moment is argued not to be determined by the actual drug dose, but by the dose the organism expects. Model simulations show the compensatory response to be the primary factor in curing diseases while it can be isolated from the drug effect to cure without the side effects characteristic of drugs. If instead of homeostasis a better functioning model had enabled a real understanding of the body’s defense mechanism, the compensatory response could have been a powerful tool of modern medicine.

## 1. Introduction

The general opinion in medical science is that bodily dysfunctions can be satisfactorily treated by drugs. Medical science uses a mathematical model to help to understand the effects of drugs: homeostasis. As will be discussed, homeostasis is too simple a model to be of much use for understanding the effects of drugs on the physiological processes in the body. As a consequence, drugs are developed experimentally through trial and error, without much insight into the way they influence the basic functioning of the physiological processes they are intended to correct [1–3].

A fundamental problem in this respect is that a dysfunction of the body is difficult to define. Usually, a disturbance in, or a failure of, an organ of which the symptoms are most pronounced is assumed to be the cause of the patient’s complaint. But all regulations of processes in the body are strongly entwined and a dysfunction of one process is nearly always accompanied by dysfunctions of other processes.

All physiological processes in the body are regulated by feedback. Within feedback loops, there are of course processes that are in themselves not regulated, but the overall feedback loop within which they function always controls their output. Within a feedback system, all activity is interlocked and it is fundamentally not possible to predict the effect of an action somewhere in a feedback loop on the loop’s total functioning, other than with a mathematical model [4–6]. However, as there is no functioning mathematical model of physiological regulation in use – as I will discuss in depth – the cause of a dysfunction of the body can seldom be ascertained with much certainty. In addition, physiological regulations are immensely complex, while both the regulation of individual processes and the overall regulation are essentially non-linear with threshold values, hysteresis and distinct adaptation processes with widely different time constants and in many cases fundamentally different tasks in the organs and in the body as a whole. Consequently, insight into the exact nature of a certain dysfunction in the body is difficult and often impossible to establish.

In this paper, the shortcomings of the current approach to treating diseases with drugs will be discussed while the benefit of using the compensatory response of the body’s adaptation mechanism in treating diseases will be expounded on.

## 2. Drugs and their effects

Outside of mechanical interventions, medical treatment is largely based on the administration of drugs, called medicines in medical science. However, due to the extensive interconnection of the processes and their regulations in the body, correcting a dysfunction of the body with drugs will always affect the overall functioning of the organism in an unpredictable way. A dysfunction of a certain process is generally accompanied by a dysfunction of other processes. They provide the information on which the process bases its functioning. If one tries to determine the cause of the dysfunction of those other processes, one is confronted with comparable complicating interactions, and so on. In addition, it is not possible to identify the cause(s) of a dysfunction within a regulated process with any certainty because in a feedback loop processes are completely interdependent so that correcting the output or functioning of one, or part of a, process never only corrects the given dysfunction but also changes the other functions in the loop.

The general understanding in medical science of how drugs work is largely based on a hundred-year-old mathematical model, homeostasis, introduced by Walter Cannon in 1929. A mathematical model is an expression of the processes it is intended to describe. The theory about how those processes function, on which the model is based, can then be tested by comparing simulations with the model with the situation in vivo. Having a working mathematical model of a physiological process means that there is a certain level of understanding about the functioning and behavior of that process. The model can then aid research into the applicability of the theory and the model in different situations and can predict the behavior of the process. When the simulations do not correspond to the behavior of the physiological process, either the theory is not sound, or the modeling is inadequate. The quality of this research consequently depends largely on the quality of the theory and the ability of the mathematical model to express and predict the behavior of the process in different circumstances. In a series of papers on adaptation and drug tolerance, I argued that the mathematical model of homeostasis cannot describe the effects of drugs in any acceptable way and presented a model of intermittent adaptation which is much more realistic in its description of the behavior of physiological processes [4,5,7–13]. Simulations with this model show a clear resemblance to the known in vivo reaction of physiological processes to disturbances.

## 3. Homeostasis and health

Homeostasis – the name given to the mathematical model used in present day medical research – keeps the regulation of a physiological process functioning at a fixed level by feedback. In previous research, it was expounded that physiological regulation does not regulate processes at a fixed level, but lets them function optimally by adapting to changes in circumstances. Feedback in itself is not adaptive. In physiological regulation, however, adaptation and feedback are inseparable components, together determining the behavior of physiological processes and the body as a whole. The presence of adaptation in the feedback loop makes physiological regulation extremely complex and very different from just feedback.

With regard to ‘optimal,’ we cannot know what criteria define optimal for a certain adapting physiological system. These criteria are determined by deliberations too complex to understand and depend on the situation, on the task of the regulated system, the functioning of the organism it is part of, its history, cognitive factors, etc. Any adapting physiological system has to satisfy multiple, often conflicting, goals [4,6,12–15]. Satisfying multiple goals at the same time is incompatible with regulating processes at a fixed level.

Rather than maintaining a steady state, as Cannon proposed, living organisms are constantly striving for the best obtainable compromise in their functioning in constantly changing circumstances. An optimum for a healthy organism as a whole is a complex balancing of the importance of very different functions and processes. The outcome is not predictable and fundamentally eludes our understanding.

Health in this respect is a difficult to specify concept [16–20]. Rather than defining health to be a statistically normal functioning of the body or the absence of disease, I prefer to define optimal health as the state the body is in at a certain moment if all its processes and functions are optimally adapted to its specific environmental situation. This automatically implies that all functions of the body cooperate optimally, as noted above. It does not imply that all activity in a healthy person is in an optimal state. An animal in its natural, non-changing environment has developed optimal adaptation to all environmental influences, generation to generation, and might be defined as fundamentally healthy. The human situation is far removed from that natural state. Health in humans is consequently not more than a relatively optimal adaptation to an unnatural, continually changing situation [6,21,22].

## 4. Adaptation

Adaptation is the way living organisms deal with changes occurring in their environment. As such, it is a prerequisite for life: without adaptation to a changing environment, a living being cannot exist. Adaptation allows an organism to keep functioning when its situation changes, which implies that it solves the problems these changes create.

The development of tolerance to a drug is a good example of how adaptation develops. During the repeated but relatively short-lasting presence of a drug the body is not accustomed to, it slowly learns to oppose the drug’s disturbing effects: every time the problem occurs, the organism thinks it over and finds a solution on the basis of knowledge gathered on previous occasions. This learning process in drug tolerance is initiated when the drug is detected by the gustatory system – the natural way – or by environmental clues the organism learns to associate with the drug’s presence.

Adaptation to drugs may be seen as an intermittent process of learning by the organism: during each disturbance, it progressively learns how to deal with the recurrent change in its internal environment to keep functioning optimally. When full adaptation is established, the organism has learned to respond as effectively as possible in the given circumstances. Adaptation to changes in circumstances always takes place; a change in drug dose creates a new situation for the organism, which requires the processes involved to adapt to a new mode of functioning. See Section 7.1.

Adaptation is an integral part of any physiological regulation. The physiological adaptation mechanism itself is a feedback loop (see [4,6]). This implies that one cannot separate adaptation from feedback. Physiological regulation is nearly always adaptive, which largely determines the behavior of the regulation. At the same time, almost every regulation in the body is influenced by other regulations, which makes physiological regulation an even more complex mechanism and the reaction of a particular regulated process to a disturbance extremely difficult to assess. Homeostasis assumes regulations in physiological processes to consist of feedback only. It can consequently not describe the true behavior of physiological regulation in any way, as feedback itself is not adaptive.

## 5. The history of homeostasis

Homeostasis was introduced a hundred years ago by Walter Cannon [23], but the idea for it came from Claude Bernard, a French physiologist, who in 1878 wrote [24] cited by [23]: ’*It is the fixity of the “milieu interieur” which is the condition of free and independent life. All the vital mechanisms however varied they may be, have only one object, that of preserving constantly the conditions of life in the internal environment*.’ Cannon’s contribution was that he thought he could show by a host of examples that the processes in the body are kept constant by feedback: the functioning of the body is in a ‘steady state’; their conditions are stable and held constant by means of feedback.

However, feedback, or control theory in general, is difficult or, more precisely, it is incomprehensible: the behavior of a feedback system cannot be understood by means of qualitative analysis; it needs to be analyzed quantitatively, for instance by a suitable computer program. But regulated processes in living organisms are extremely complex with a great many parameters influencing those processes, while calculating the behavior of a regulated system is only possible if there are enough data present about the factors affecting the regulation.

In Cannon’s time – the early 20th century – research on feedback was in full swing. It led to procedures for the design of technical feedback systems – mostly graphically as computers did not exist then – and to methods for predicting their behavior. The pioneering work of Nyquist, Bode, Laplace and Guillemin is still prominent today [25–28]. Feedback became a hit and led to the realization that the functioning of every process in a living organism is regulated by feedback. In medical science, feedback became homeostasis: physiological processes are kept functioning at a constant level by feedback. Homeostasis has been the basis of influential theories like systems theory by Bertalanffi and cybernetics by Norbert Wiener, which proposed that physiological processes could be simulated by electronic feedback models [29–31]. In the mathematical models of drug tolerance developed on the basis of these theories, the effects of drugs are assumed to be counteracted by a feedback mechanism which keeps the processes involved functioning at a preset level [32–40].

With feedback, research into the behavior of regulated processes in the body suddenly became part of what was then modern science. That Wiener, with his technical background, believed in it is not surprising. In the war, he worked on the control of firearms, with feedback of course, and there it did apply. That technical and physiological regulations are totally different, he apparently did not realize.

Wiener did try to simulate the behavior of homeostasis and for a single cycle of a recurrent disturbance he could show a signal shape assumed to be characteristic of the process. The limitations of the result were not the fault of Wiener; he just did not have the means to examine the behavior of physiological processes better with the tools at hand in his time. The computer did exist then, but it was analogue and used vacuum tubes for the computations. A model of a simple feedback system was difficult to get stable during one cycle of a periodically occurring disturbance (see e.g [41].). And as one cycle of a series of disturbances does not give information about adaptation – an essential part of any physiological regulation, as discussed – the model of homeostasis was not really tested. While modern digital computers have turned the modeling of extremely complex processes into common practice, nobody extended Wiener’s work to investigate the real behavior of physiological regulation. Simulating the behavior of a physiological process with a simulation program on a modern computer will immediately make clear that homeostasis does not describe physiological processes in any accurate way.^a^ I have extensively elaborated on this subject in previous publications (see e.g [4,13]).

**Figure 1.**
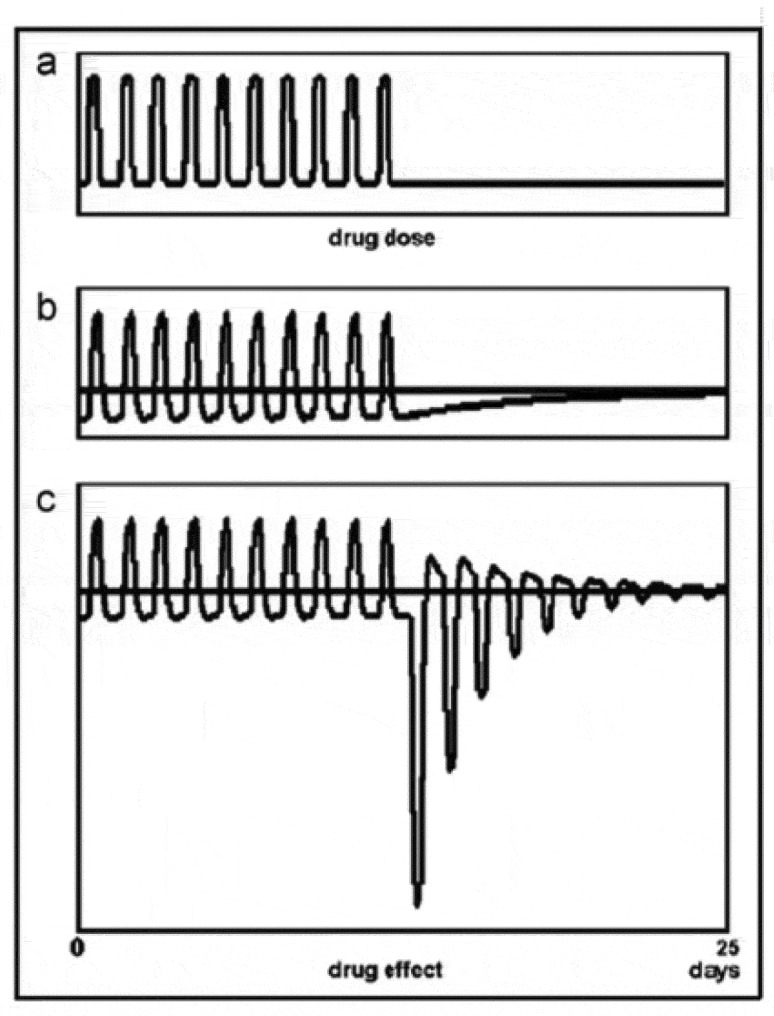
Simulation of the effect of abrupt drug withdrawal in tolerant (b) and dependent (c) subjects. The drug is administered once a day.

Figure 1 shows a simulation with the mathematical model published previously (see appendix). The simulation represents an abrupt discontinuation of a medication in tolerant and dependent subjects. In dependent subjects, the reactions following withdrawal can be extremely strong. In just tolerant subjects, the negative state after stopping a medication is comparatively small, but it represents a worsening of the symptoms the medication counteracted and is a strong motive to keep using the drug.^b^ The complex behavior of physiological processes in response to successive drug administrations shown in the figure cannot be simulated with the model of homeostasis, as will be discussed below.^c^

## 6. A functional model of physiological regulation

As my previous work demonstrates, a working model of the complex physiological regulation process has to satisfy a series of essential preconditions:

1. Any drug evokes a response in the organism which opposes the action of the drug: the compensatory response ^d^
2. The effect of a drug decreases in time when the adaptation mechanism learns to match the compensatory response to the disturbance^e^.
3. The magnitude of the compensatory response to the administration of a drug, or more generally to a disturbance, is not determined by the actual magnitude of that disturbance, but by the magnitude the organism expects, or is used to [4]. Medical science is not really aware of this phenomenon, but it is a fundamental part of the mechanism and largely determines the drug effect.
4. The compensatory response is triggered when the drug is detected in the natural way by the gustatory or olfactory mechanisms. When that information is not available, for instance because the drug is administered intravenously or in pills or capsules, the body uses other information, like the time of day or the environment where the drug is taken, to trigger the compensatory response [42,43,44,45].
5. The decrease in drug effect in response to a repeated administration of the drug ultimately settles at a level determined by the open loop gain of the regulation loop (the ability of the process to suppress disturbances).^f^ The open loop gain in physiological regulations is very small, with values of 4 to 6 being typical (see [11,13]).
6. Due to the indirect relation between the actual quantity of the drug and the action of the adaptive mechanism, reactions to a change in dose can be large, depending on factors such as the level of tolerance, dependence or addiction (See Section 7.1),^g^.
7. There are two ways in which drugs may disturb physiological processes: a) a drug changes the level of a regulated substance in the organism, increasing it by its presence when it is similar to the substance in question, or decreasing it, for instance by neutralization. b) a drug disturbs the information transfer in the organism. These two possible effects of drugs have essentially different implications as was discussed extensively in previous papers (see e.g [4, 5].^h^
8. The reaction of an adaption process to a drug consist of two distinct processes: adaptation to the effect of the drug and adaptation to the interval between drug administrations. For the organism, the beginning of the drug action and its ending constitute different disturbances because they are the beginning of different, opposite events: the drug effect and the interval between drug-taking. Consequently, that a process is adaptive necessarily implies that there is no base line as its functioning at any moment fundamentally depends on the situation (see [4]).

In addition to all these characteristics, an adequate model of the adaptation process has to consist of two autonomously functioning regulatory systems: a complex non-linear dynamic system which minimizes the direct effect of a drug on the regulation, and a slow system which minimizes the effect of the remaining disturbance. The latter regulation system has a relatively long time constant. The dynamic regulation system has a short time constant and consists of the two systems mentioned above in (8): one to adapt to the effects of the disturbance and one to adapt to the interval between the disturbances.

Satisfying all these prerequisites requires a complex model (see the appendix), but they are fundamental for any realistic model of physiological regulation. Homeostasis does not meet any of the above characteristics of a regulated physiological process, however, other than the feedback component and it should be clear that homeostasis is not usable in any way as a model to describe the functioning of physiological processes.

In addition, and this is an extremely important point, the behavior of even the most simple feedback system cannot be discussed and understood in a qualitative way; it has to be established quantitatively. From a previous paper [5]: *The importance of conducting research into the behaviour of regulated physiological systems using control theoretical principles cannot be overemphasised as the behaviour of a regulated system can only be understood from the behaviour of a mathematical model describing it. Even the behaviour of the simplest regulated system cannot be described other than mathematically. The behaviour of more complex regulated systems can only be understood from simulations with computer programs using advanced, iterative methods to solve the differential equations involved. This implies that a model which is qualitative only may never involve feedback systems as their behaviour cannot be predicted* .^i^ In explaining homeostasis, physiological regulation is often compared to the temperature regulation of a room by a thermostat [99]. However, comparing the enormously complex physiological regulation mechanism to just about the most simple technical feedback system completely overlooks all the important characteristics of physiological regulation as listed above .^j^

When modern computing is used to establish the adaptive behavior of a physiological process, it becomes instantly clear that homeostasis does not describe the characteristics of the process in any acceptable way. For instance, any computer simulation immediately demonstrates that feedback alone (homeostasis) cannot simulate the decreasing effect of a repeatedly administered drug of constant quantity.^k^

## 7. Drug dose and drug effect

### 7.1. The dose-effect relation

The oral administration of a drug is the primary and natural stimulus for the development of tolerance. For a living organism, there is a clear relationship between oral drug-taking and the drug effect; after all, the natural route of an exogenous substance into the body is through the mouth. The primary functions of taste and smell are there to allow the organism to recognize a substance when it enters the body, enabling it to anticipate its effect and to take appropriate measures in time.

Adaptation to disturbances from exogenous substances is fundamental for any living organism and can also be observed at cell level, where the mere presence of a drug can induce tolerance without the mediation of higher structures like the central nervous system [6,46–49]. This has been demonstrated explicitly in isolated cell cultures, where repeated stimulation with toxic substances or changes in temperature induce tolerance [9]. That the organism’s response to the effect of a drug is triggered by the oral administration of the drug is clearly demonstrated by, for instance, the oral administration of glucose, which almost immediately results in an increased release of insulin into the bloodstream [32,50,51, 52,53,54]. The organism will make use of any cue it can find to anticipate disturbances of its functioning and the oral process in drug-taking seems essential in this mechanism.

While a drug’s chemical properties determine which processes are disturbed, it is the quantity of the drug which determines how much those processes are disturbed and hence the extent of the measures the organism must take to reduce the drug effect. This quantity, however, cannot be determined at an early stage. The organism is, for example, unable to determine the quantity of a medication before it is dissolved completely, or whether a cup of coffee is followed by a second or third. Such information becomes available only after a relatively long time, which is (or may be) too long for the processes involved to counteract the drug’s disturbing effect in an effective way. When the dose of a drug changes, the organism possesses in the first instance no definite information about the magnitude of the measures it has to take and continues its normal routine. It consequently bases the magnitude of its reaction on the drug dose it expects: the usual or habitual drug dose. In practice, this will be about the average dose of a number of previous drug administrations. It follows that tolerance to a certain drug does not mean that the organism knows how to cope with that drug, but that the organism knows how to cope with a certain quantity of that drug only. A change in the habitual drug dose will therefore result in a period of incomplete tolerance during which the effect of the drug on the organism differs substantially from the tolerant situation. The functioning of the organism will then remain disturbed until it has learned to cope with the new drug level and has become tolerant to the new drug dose. This period of incomplete tolerance after a change in dose is a direct consequence of adaptation, which is fundamentally a slow process; the body slowly has to learn how to deal with a new situation.

**Figure 2.**
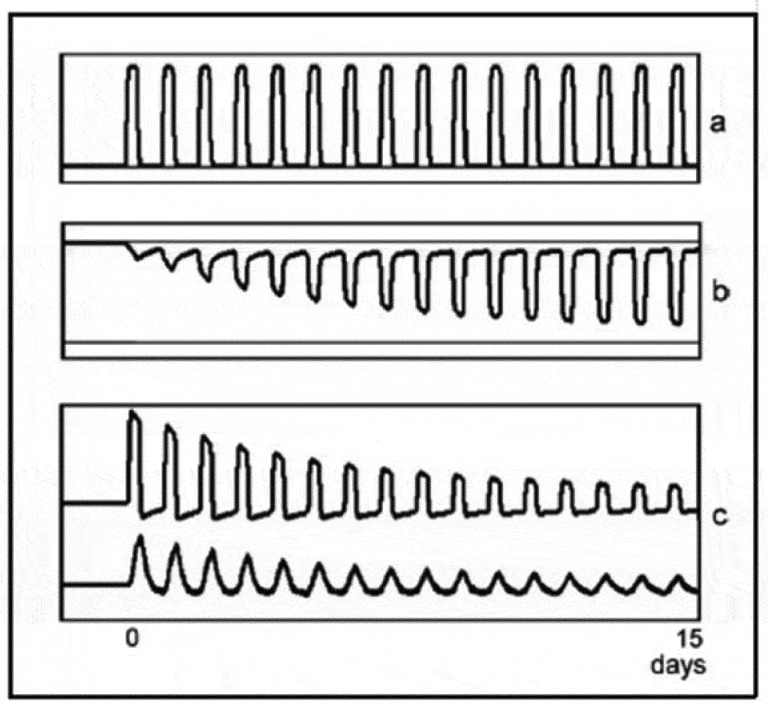
a) the exogenous substance when it enters the bloodstream. b) compensatory response during tolerance development c) both effects added and the resulting blood level.

In Figure 2, a simulation with the mathematical model shows different components of the adaptation process to an exogenous substance. What becomes clear from the figure is that the magnitude of the compensatory response (Trace b) can considerably exceed the effective drug effect (Trace c). This phenomenon has large consequences when there are changes in drug dose.

### 7.2. The effects of a change in drug dose

**Figure 3.**
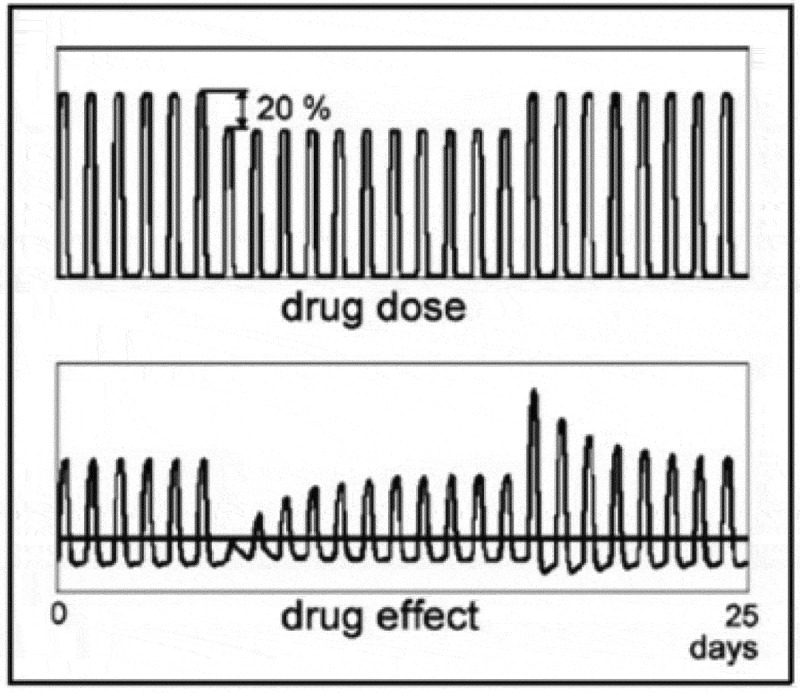
A simulation of the effect of a small change in drug dose after tolerance has developed.

Figure 3 shows a simulation of the effect of a small change in drug dose after tolerance has developed. For a given set of parameters, a 20% decrease in dose results in an initial suppression of the drug effect. An increase in dose back to the original value causes an initial comparably large increase in the drug effect.

This remarkable reaction of the body to changes in drug dose has far-reaching implications. In medical science, this phenomenon is not understood and is generally considered paradoxical. However, there is no such thing as paradoxical behavior on the part of the body. It is not a very helpful way of describing a poorly understood situation. This lack of understanding results primarily from the poor model used to describe the behavior of the body. With just feedback – homeostasis – it is impossible to explain how the body manages the difficult situation where it does not receive information about the drug dose in time. Feedback regulates the output of a process at a level determined by its reference value. But feedback completely fails to account for the often extreme effect of changes in drug dose.

**Figure 4.**
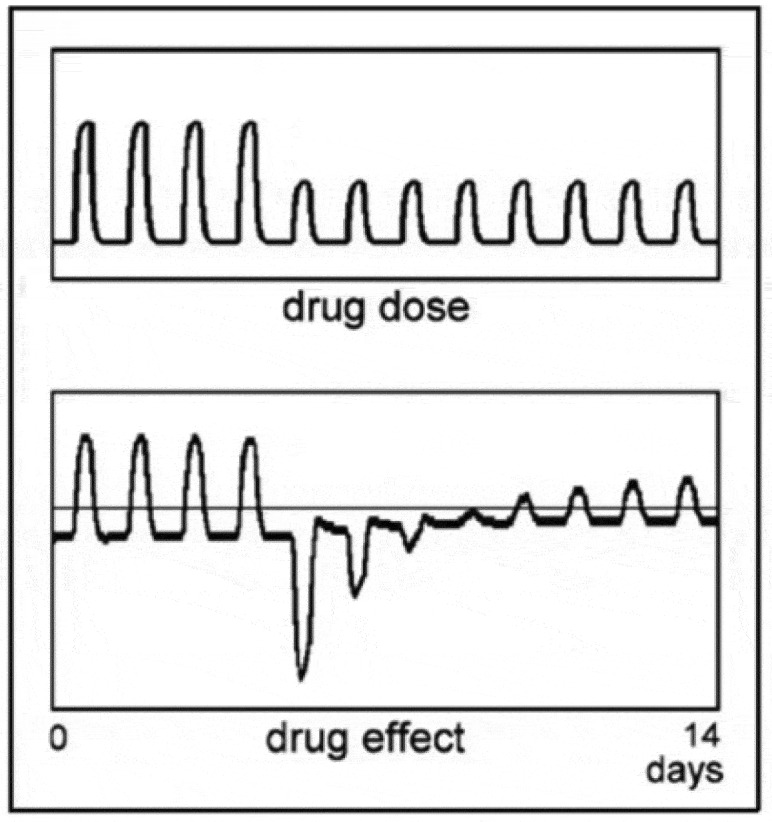
Effect of reduction in drug dose to 50%.

If the body expects a drug dose much larger than the actual dose, the compensatory response will exceed the direct drug effect, resulting in an effect opposite to the normal drug effect. For pain medication, for instance, it means that the pain gets worse .^l^ Figure 4 shows a simulation of this situation: the effect of a reduction in drug dose to 50% results in an initial negative drug effect.

### 7.3. Reducing the drug dose

**Figure 5.**
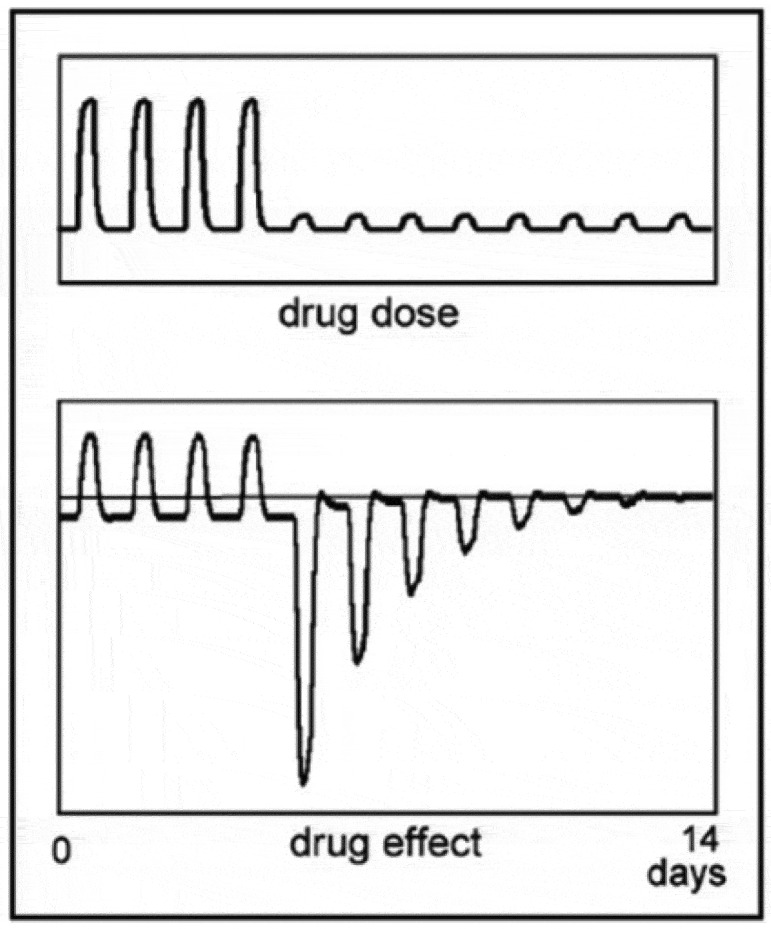
Effect of reduction in drug dose to 10%.

When the dose is reduced even more than the 50% shown in Figure 4, the net result will be approximately the compensatory response alone, as is shown in Figure 5, where the dose is reduced to 10%. When the drug dose is reduced even more, but the drug is still recognized by the body, about the same negative effect will occur, as the contribution of such a small dose to the total drug effect becomes negligible. When, in Figure 5, the tolerance mechanism slowly adapts to the small dose, the compensatory response becomes proportionally small. These simulations with the mathematical model demonstrate that the initial effect of a change in drug dose can be significant, which reveals a very different relation between drug dose and drug effect than is generally assumed. The magnitude of the compensatory response to information from the past about a drug can in most situations not be predicted as it will depend on the history of the particular process and its possible dependence on external stimuli. In the literature on the theory of Pavlovian conditioning, many examples are given of reactions after drug use has stopped for a long time [42,45,55]. But the activation of the compensatory response by paired stimuli in Pavlovian conditioning is only one part of the complex way in which the compensatory adaptive mechanism may be triggered. This mechanism will respond to any information it can gather about the presence of a drug (see e.g. [56]). Paramount is the oral detection of the drug in the natural, oral route: as long as the drug is recognized if taken orally, any dose may trigger the compensatory response to that particular drug. It is worth bearing in mind that the mouth is an extremely sensitive sensor; it recognizes very small doses of substances, down to 0.1 ng/l or less (see e.g [57]).

**Figure 6.**
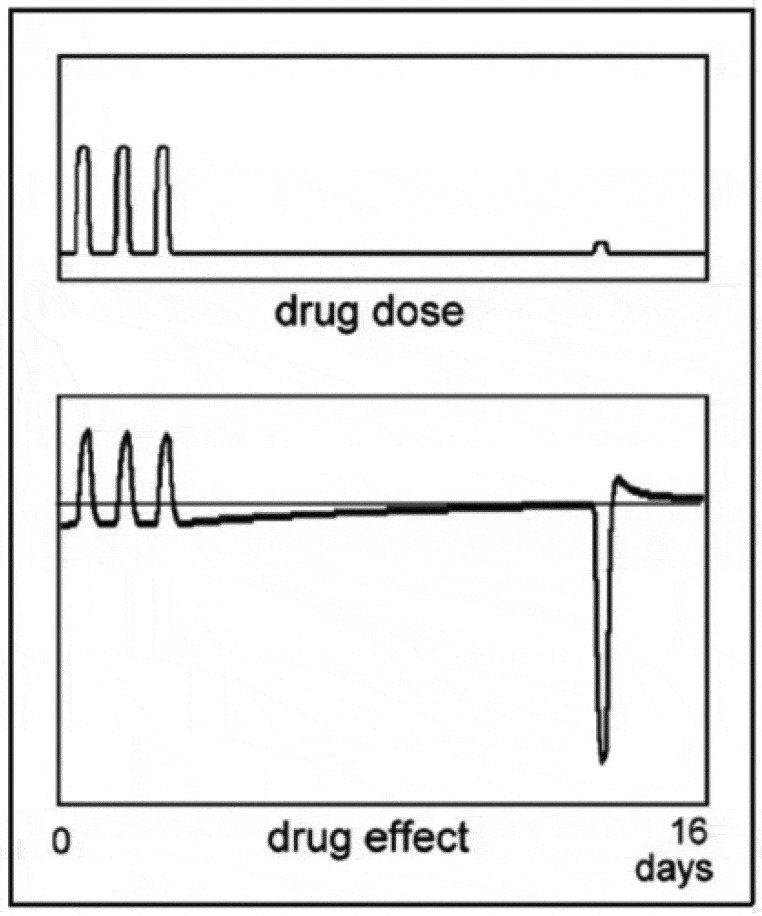
A simulation with the mathematical model of the effect of a small dose of a drug, administered at an arbitrary time after the administration of the drug to which tolerance has developed is discontinued.

These large reactions to a small drug dose are not restricted to a sudden change in dose, but will show on every occurrence of a small drug dose when the body has developed tolerance to a larger dose. This is demonstrated in the simulation in Figure 6, where the small dose is administered at an arbitrary moment after the regular administration is stopped (see [11,13]). As long as there remains adaptation to the drug or better still, the adapted situation is remembered, a small dose may evoke a reaction. For natural substances, the body generally has a certain level of tolerance, which implies that a small dose of a natural substance may cause a reaction when the body expects a larger dose. What that expectation is based on will depend on the nature of the drug, the particular individual concerned and other aspects of the situation.

## 8. Curing disease

### 8.1. The compensatory response

Medicines are usually administered to cure a disease or to suppress its symptoms. If drug A is assumed to be curative because it removes or reduces the symptoms of a certain disease, its curative action lessens in time through adaptation by the body: the effect of the drug is opposed by the compensatory response the drug incites. As the compensatory response is opposite in its effects to the drug effect, the compensatory response’s action itself worsens the disease. That the compensatory response to a drug exacerbates the patient’s diseased state is an inevitable consequence of the disturbing action of drugs (see [4]).

In this context, it should be mentioned that concepts like illness, disease and especially curability are often defined in very different ways. On the one hand, there are those who consider present medical practice as curative, in spite of a low effectiveness and accompanying side effects of the medicines used; there are others who largely reject its effectiveness altogether [58–63]. See also Section 3 on health.

If a hypothetical drug, let’s say, drug B, excites patient symptoms which are exactly the opposite to those of drug A and as a result worsens the disease and/or its manifestations, the compensatory response drug B generates lessens these symptoms. If the patient has developed tolerance to drug B, his compensatory response will, consequently, oppose the symptoms of the disease and may be curative for the same reasons drug A is assumed to be curative.

The most important difference between the curative power of drug A and that of the compensatory response to drug B is then that, while the dose of drug A has to be steadily increased due to the adaptation which develops – a permanent complication in drug prescription – no adaptation to the compensatory response to drug B will develop: a compensatory response does not evoke a compensatory response .^m^

### 8.2. Curing disease by aggravation

A compensatory response to a drug which evokes symptoms similar to those of the disease might have a curative effect. In Figure 1(b), it can be seen that when tolerance to a drug has developed, withdrawal of the drug will result in symptoms opposite those of the drug effect. .^n^ This after-effect has been used throughout the ages to cure people of diseases. High doses of a drug which aggravate the diseased state of a patient are given, and when the drug is withdrawn, the reaction which follows often cures the patient. If the patient survived the treatment, that is. What happens is that the reaction of the body after the drug is withdrawn opposes the diseased state, as can be seen from the figure, constituting a curative effect. To obtain a satisfactory reaction, the dose of the drug has to be large, with all the suffering that entails. If the patient then dies, it is assumed he died of his disease, as the symptoms the drug evoked strongly resemble those of the disease.

A well-known example of this practice is Schubert, who died of syphilis, history teaches us. But he had been treated against the disease with large doses of mercury. Whether he died of mercury poisoning or of syphilis cannot be determined [64–68]. Clarke [69]: *Mercury so far corresponds to syphilis that many undoubted cases of mercurial poisoning have been diagnosed by experts as syphilis*. The same is seen in malaria patients treated with large doses of quinine, the poisoning symptoms of which largely resemble malaria symptoms such as paroxysmal fever [68,70,71]. When the patient develops tolerance to that drug, the malaria might be cured after the treatment is finished. And much the same happens today with cancer treatment which uses large doses of strong carcinogenic substances [1,72–76] with all their terrible side effects. When the patient survives, he has been saved by the treatment; when he dies, he dies of cancer. Little has changed over the centuries.

The treatments in these examples resemble each other in that they determine a cause whose elimination is assumed to cure the patient: the bacterium in syphilis, the parasite in malaria and cancerous cells in cancer. What really happens in cancer treatment is explained above: the reaction of the body after the treatment with cancerous drugs is stopped is curative and may cure the patient [77–79]. From Figure 5 it can be learned that when the cancer therapy is finished – all cancerous cells have been killed – a short continuation of the therapy with small doses of the drugs used may considerably enhance the chance of a restoration of health because of the large compensatory responses – opposite to the drug’s cancer symptoms – evoked that way.^o^

Although the poisoning symptoms of the drugs used in this kind of treatment often remarkably resemble those of the disease, this resemblance is still limited, as evidenced in the great many cases where cures brought about in the above fashion proved to be incomplete or temporary. If a hypothetical drug causing the same symptoms of the disease on all levels had been employed, the body’s compensatory response might have cured the patient completely. Or to put it differently: a compensatory response which accurately matches the whole symptom complex of the disease will remove the symptoms and consequently restore the organism’s original state of functioning.

### 8.3. Common drug treatment

The usual way of treating a patient is by administering drugs which oppose the symptoms. While the initial effect of those drugs is generally satisfactory for the patient, what follows is problematic. Drugs that oppose the symptoms of the patient evoke a compensatory response that opposes their effect and consequently worsens the patient’s symptoms: after the treatment, the disease has got worse. No permanent cure can be procured this way, in contrast to the curative potential of for instance mercury for syphilis after the treatment is finished. The accompanying adaptation to the drug furthermore necessitates a progressive increase in drug dose with further complications and may ultimately result in total dependence on the drug or even addiction to it. Using medicines in high doses to counteract a dysfunction of a process inevitably interferes with the regulation of processes whose parameters and reference values depend on or are influenced by the functioning of the process the medication is aimed at. The consequences of this disruption of the organism cannot be predicted. Every drug affects processes which are not involved in the dysfunction of the targeted process [4,6]. The potentially complex situation that develops as a consequence can be very harmful to the patient, but is then commonly treated with other drugs which in themselves will cause comparable disruptions of other processes. Another problem is that the complex action of a drug in an individual is not known, other than from the extremely crude symptoms which are designated the drug effect. But, in most cases, how all the alterations in bodily processes caused by a drug accumulate to these observable changes cannot be ascertained with much certainty. Using drugs to heal the body of undesired functioning generally causes changes which just add to its existing dysfunction.

With prescribing cascade, the medical profession has tried to manage the consequences of medicine use [80–83]. Its effect is however an increasing dysfunction of processes in the patient. Side effects of medicines are not accidental; all drugs have multiple effects. What processes a certain drug may disturb in a patient is usually not predictable. A drug may cause disruptions of the organism which may become manifest only after a long time, when the relation with the drug is not apparent anymore. Medicines affect the body in a generally unpredictable way and the practice of suppressing unintended effects with other drugs makes the situation even worse.

### 8.4. Curing disease with the isolated compensatory response

When a drug generates symptoms in a patient very similar to those of his disease, the reaction after the treatment may cure the patient, assuming his health has not deteriorated too much during the treatment. The latter is the real problem: while the reaction after the treatment might be curative in that it boosts the body’s own defense to the disease, this healing effect is often impeded by the treatment’s disastrous attack on the patient’s health. It is, however, possible to activate a compensatory response isolated from the troublesome effects of large drug doses as is made clear by the simulation shown in Figure 6, where the compensatory response is evoked by a small dose of the drug, long after its direct effect has ceased. Although the compensatory response is essentially a reaction of the body to the effects of disturbances, it can apparently be generated separately from the disturbance it is intended to oppose. This phenomenon is a consequence of the body’s problematic relationship with the actual magnitude of the disturbance. As discussed in Section 7.1, the magnitude of the compensatory response does not depend on the magnitude of the actual disturbance but on the magnitude the body expects. While the compensatory response in Figure 6 is activated by a small drug dose, it might also be evoked by an environmental cue paired to taking the drug in the past and there are probably several more ways in which this can be accomplished.

Generally speaking, when there exists tolerance to a drug, a small dose only triggers the compensatory response, resulting in effects opposite to the usual drug effect. A small dose of a drug apparently isolates the compensatory response from the drug effect, which seems a peculiar phenomenon. But as this reaction of the body to a small dose is a fundamental characteristic of the way the body reacts to changes in drug dose, it must be part of any comprehensive mathematical model of physiological adaptation, as observed in earlier research (see e.g [4,5,13]). With a small dose, the body reacts to information it receives about a drug which effectively may not be there in any substantial amount. The information about the drug presence is what is central in this situation: if the body assumes that the drug is present, based on whatever information, its reaction, the compensatory response, will be based on the drug dose it expects.

The above has made clear that small doses of drugs showing the same symptoms as the disease can elicit an isolated compensatory response which can cure the patient. Apparently, the whole terrible routine of giving drugs in large doses is superfluous as it is only the compensatory response which can bring the body back to its normal functioning, while this compensatory response can be evoked in isolation, without the drug effect that normally triggers it.

### 8.5. Curing diseases with homeopathy

A small dose of a drug which is still large enough to provide the organism with information for recognizing the drug will evoke a compensatory response whose magnitude is based on the dose the organism assumes or expects it to be. This reaction may then be significantly larger than the dose warrants. The magnitude of the compensatory response depends on the level of tolerance developed for the drug, but it may originate in a memory of a drug dose ingested long ago or in innate information. This possibly large reaction of the body to a small dose of a drug inevitably leads to homeopathy.

In homeopathy, small doses of drugs are administered which, in larger doses, would generate a symptom complex similar to that of the patient. The compensatory response evoked this way then opposes the symptoms of the patient and may restore his original state of functioning. The drugs in homeopathy are chosen to oppose as many as possible of the symptoms displayed by the patient. For this purpose, the effects of most substances known in nature have been investigated in high doses. The result is a large materia medica of symptoms of natural poisons and other substances which affect the human body, with the symptoms classified in terms of their intensity and probability of occurrence.

This paper demonstrates that homeopathy makes use of the normal effects of drugs on the body and medical science might be expected to consider it a perfectly scientific way to cure diseases. This is however not the case. The attitude of medical science toward homeopathy is extremely negative. Why that is the case might well be the result of extreme prejudice, defensive thinking or group dynamics. But the chief cause is the model underlying medical science, homeostasis, which cannot explain the effects of small doses of drugs. If a better functioning model had enabled a better understanding of the vital role played by the compensatory response in the body’s defense mechanism against exogenous substances, the small dose could have been a powerful tool of modern medicine.

Before the second world war, the dispute between homeopathy and what today is called conventional medical science was much more balanced. Homeopathy was flourishing and in the Netherlands alone there were three homeopathic hospitals, while England had five, with the English royal family as its patron up to this day. The widespread use of antibiotics after the war changed the scene dramatically as it was generally believed that they would provide an easy cure to every infectious disease. That they did not and that today their use is often recognized to be problematic (see e.g. [84]) has not changed the negative attitude toward homeopathy which had in the meantime developed in medical science. The faith in the blessings of conventional medical science is also strongly boosted by the fast and sweeping development of medical technology suggesting a medical quality which is simply not there.

### 8.6. The high dilution dispute

Homeopathy has always largely been an empirical method. Hahnemann, the founding father of homeopathy, developed his method step by step, solving the many practical problems when they presented themselves. After discovering the curative effect of small doses, he was confronted with the problem that, after diluting a substance a number of times, further diluting did not work well. It then appeared, probably by accident, that if he shook the substance vigorously with every further dilution, it kept its desired effect. He assumed that certain powers in the substance were freed that way and consequently called it dynamisation or potentization: shaking exposed the true potency or power of the substance.

At present, there are several theories in homeopathy trying to explain the phenomenon. One way or the other the information about the drug’s properties appears to be transferred or retained with every subsequent dilution. However, whatever the workings of very high dilutions, the value of homeopathy should not be made dependent on a non-essential part of the method whose functioning has no definite explanation as yet. There are many phenomena in science which are accepted although not understood.

There is no fundamental difference between the effect of low and high dilutions in homeopathy. The emphasis put by critics of homeopathy on the incomprehensibility of the action of very high dilutions is not justified. Homeopathy functions well with dilutions where information about the drug is gustatorily detected by the body in the normal way and that should be the subject of discussion. The potential effect of small doses of drugs as discussed above sufficiently supports the validity of homeopathy as a therapeutic method. The effects of high dilutions should be accepted as an apparently empirical fact which is in no way decisive for the therapeutic system.

## 9. Discussion

This paper argues that homeostasis does not describe the characteristics of regulated physiological processes, other than the feedback component, and the model of homeostasis should therefore not be used to describe the functioning of complex physiological processes. Nevertheless, homeostasis is the only mathematical model medical science knows. The consequences for the practice of medicine are disconcerting as apparently it has no functional model to describe the effects of its medicines. The medical profession exposes their patients to the disrupting effects of drugs without any fundamental knowledge of how the drugs disturb the physiological processes in the body, while the drugs are generally administered in large quantities. Medical science and pharmacology have no understanding of the relation between drug dose and drug effect and what happens to the body when it is disturbed by a drug. Even the dose-response curve used in pharmacology is invalid, as I demonstrated in previous papers [11,13]. The model used by medical science is completely outdated and based on an extremely simple theory which is never really tested and which cannot in any way explain the complex processes taking place. Homeostasis cannot explain even the decrease in effect over time of repeated drug administrations, a major disruptive phenomenon in drug-based therapy, something I addressed in previous publications (see e.g [4,13]). One might object that in medical science adaptation is assumed to be a phenomenon independent of homeostasis. However, medical science does not have a mathematical model explaining adaptation either. And, as pointed out, feedback and adaptation cannot be separated in physiological regulation.^p^

The use of homeostasis in medical science and pharmacology as the basis of research into the functioning of physiological processes makes abundantly clear that the current medical approach to treating ailing patients is failing. A science based on a nonfunctioning model is of little value to say the least. But homeostasis is easy to understand and without a functional mathematical model its assumed behavior can be discussed freely without anybody being proved wrong. In contrast, my model is complex and therefore difficult to understand and developing such a model for specific physiological processes requires a knowledge of control theory and computer programming and the ability to apply such a theoretical mathematical procedure to the analysis of the extremely complex subject of physiological regulation. These skills are not readily found in the medical field.

As it is, homeostasis is a doctrine based on assumptions which are never tested or substantiated. It tries to suggest that the behavior of the body in response to disturbances is easy to understand, but physiological regulation is extremely complex and cannot be described by this simple model. In homeostasis, insight into the real functioning of regulated processes is lacking while the different ways in which drugs influence the functioning of the body are not understood. As a consequence, drugs and drug treatments are developed on a trial-and-error basis, without a functional model guiding the process. A model of physiological regulation which in practice is qualitative only should never have been adopted as the basis of medical science, which literally determines ailing people’s life or death.

## Figures and Tables

**Figure A1.**
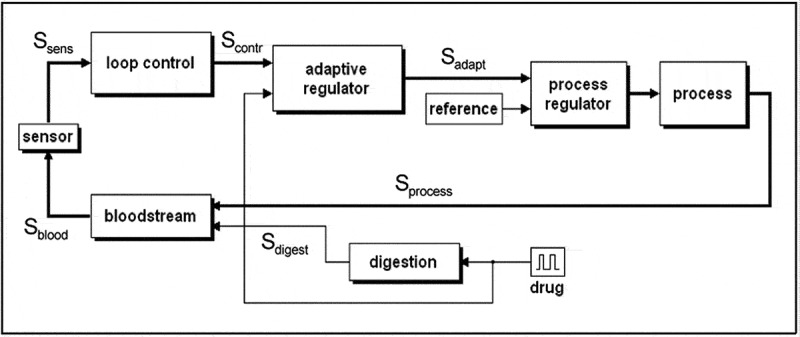
Block diagram of a regulated adaptive process.

**Figure A2.**
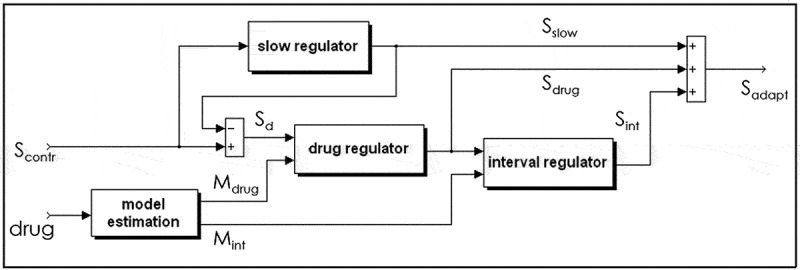
Block diagram of the adaptive regulator.

**Figure A3.**
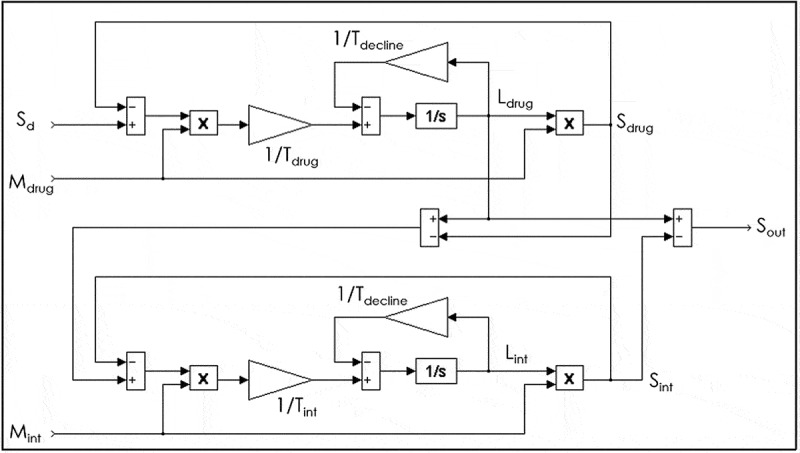
Fast regulator implemented in Simulink.

## Notes

1. Besides theories of drug tolerance based on feedback, there are theories which do not regard tolerance development as the result of a regulated process. An influential theory was developed by Solomon and Corbit: the Opponent-Process theory [92–95]. In this theory, the drug is thought to trigger a response known as the A-process. The A-process induces a reaction called the B-process, which opposes the A-process and increases in magnitude by repeated elicitation of the A-process. The A-process is fast, while the B-process is delayed and slow. As the difference between the A-process and the (negative) B-process is the ultimate effect of the drug, the drug effect will slowly decrease. Several theories are based on a model of habituation developed by Rescorla and Wagner, which attribute tolerance to a learned diminution of the response [55,96–100]. Dworkin incorporated this theory in a feedback model of drug tolerance [32]. Another influential theory was proposed by Siegel [38,39,42,78, 85–91, 94]. In Siegel’s theory, drug tolerance is assumed to be caused by Pavlovian conditioning: the compensatory response of the organism to the administration of a drug is triggered by environmental cues paired to the drug-taking. Poulos and Cappell augmented Siegel’s theory of drug tolerance by incorporating homeostasis, which was adopted by Siegel [37–39].

2. The fundamental difference between tolerance and dependence or addiction is that with the latter the triggering of the adaptive mechanism continues when the medication is stopped, while with just tolerance the triggering is coupled to the presence of the drug. In the model, the activation of the compensatory mechanism, independently of the drug’s presence, is assumed to be the essential factor determining the difference between drug tolerance and drug dependence.

3. As described, the regulation of physiological processes is essentially more complex than just feedback, or homeostasis, the name for feedback in medical science. In the rest of the paper, I will assume this to be known and, when I use the term regulation, it will always refer to that more complex structure.

4. When the paper uses the word ‘drug,’ it is meant to denote all exogenous substances which affect the functioning of the body and incite adaptation in such a way that over time tolerance, dependency or addiction develops.

5. The compensatory response opposes the drug effect. This does not imply that the opposing action completely mirrors the drug effect. In practice, there are too many conflicting factors for the organism to deal with. But the opposing effect of the compensatory response is generally adequate, as is demonstrated by the universally observable decline in the effects of drugs over time.

6. The open loop amplification (gain) is the amplification of the regulated system when the loop is opened. In electronic regulations, this amplification is very high; a factor of 10.000 is common. In biological regulations, it is extremely low; 4 to 6 is typical and it can be even lower. This implies that the effect of the regulation on the magnitude of the output of a regulated biological system will always be limited and the remaining error large. Its implications, however, can be far-reaching.

7. The compensatory response to a change in drug dose is very different from the normal adaptive response. While the adaptive mechanism as a whole is a feedback mechanism, the compensatory response to a change in drug dose might be considered feedforward. Feedback and feedforward are fundamentally different mechanisms. While feedback is a regulatory mechanism in which all action is interrelated (see Section 2), feedforward is an isolated action, basically without feedback.

8. The parameters for the simulations are set to show the effect clearly in the figures. In physiological processes, they will be different in the different processes. But the phenomena shown will always be present.

9. Medical science assumes that the behavior of complex regulated physiological processes can be discussed and understood in a qualitative way. However, that the behavior of feedback systems can only be established quantitatively rather than qualitatively has been known all along in control theory (see e.g. the work of [101, 102]).

10. The same holds for the way the temperature of the body is regulated, a much-used example of assumed homeostatic regulation. In reality, the body temperature is controlled by an extremely complex, fundamentally adaptive regulatory system (see [4]).

11. In previous publications, it is demonstrated that the response of a simple regulated system to a recurrent stimulus of constant magnitude does not decline in magnitude (see e.g [4,13]).

12. This effect is usually experienced negatively; after an initial amelioration, the pain gets worse. The opposite is just as possible but in practice does not often show as medications are given to suppress unwanted situations. If a drug were used to create an unwanted effect, the patient would experience the after-effect positively [103].

13. The compensatory response is a major force the body possesses to defend itself against external disturbances, like drugs. The body has no reason to suppress such a response. In addition, the body cannot evoke a compensatory response to its action as that compensatory response would then in turn also evoke a compensatory response, etc. This does not necessarily imply that the effect will not diminish in time.

14. This effect is generally known as the rebound. However, the expression ‘rebound’ is not really applicable in this context as the effect is merely the last part of the compensatory response after the drug effect has ceased. The word rebound is generally used for the response occurring after a pulse on a system or process with a differentiating transfer function. But that response is a phenomenon fundamentally different from the compensatory response.

15. It might be helpful not to mention to the patient that the drug dose has been reduced, to make sure a possible activation of the compensatory response by paired stimuli, developed during the therapy, remains active too.

16. There have been attempts to lessen the obvious shortcomings of the model of homeostasis [19,104–112]. A well-known alternative is the model of allostasis. Allostasis tries to explain how the body, or the brain, regulates processes to accommodate changing environmental situations [19,105,107–109,111]. However, allostasis has no mathematical model other than homeostasis to substantiate its theory which, as discussed, the model of homeostasis is not capable of.

## Appendix (from Peper, 2012)

The model is a non-linear, learning feedback system, fully satisfying control theoretical principles. It accepts any form of the stimulus - the drug intake - and describes how the physiological processes involved affect the distribution of the drug through the body and the stability of the regulation loop. The model assumes the development of tolerance to a repeatedly administered drug to be the result of a regulated adaptive process; adaptation to the effect of a drug and adaptation to the interval between drug taking is considered autonomous tolerance processes.

The mathematical model is derived in detail in Peper (2004b). In the present appendix the equations are summarised. A block diagram of the model is shown in Figure A1. For the sake of brevity, the index (t)in time signals is omitted.

### A1. The digestive tract

The digestive system plays no role in the regulation loop. Drug transport through the digestive tract is modelled as a first order function:

(1) $$S_{\mathrm{digest}}=\int_{0}^{t}\mathrm{dru}g_{\;}\mathrm{dt}-\frac{1}{T_{\mathrm{digest}}}\int_{0}^{t}S_{\mathrm{digest}}\mathrm{dt}$$

The input to the block is the drug administration, *drug*. The input signal is integrated to obtain the drug level when it enters the bloodstream, the output of the block S_digest_. A fraction 1/T_digest_ of the output signal is subtracted from the input to account for the distribution of the drug in the digestive tract. T_digest_ is the time constant of this process.

### A2. The bloodstream

After digestion, the drug enters the bloodstream where it is dispersed. In the present configuration of the model, the drug and the substance produced by the process are assumed to be identical in composition and consequently add in the bloodstream. The amount of the total substance in the bloodstream will be reduced by the body’s metabolism. The processes are modelled with a first order function:

(2) $$S_{\mathrm{blood}}=\int_{0}^{t}(S_{\mathrm{process}}+S_{\mathrm{digest}})\mathrm{dt}-\frac{1}{T_{\mathrm{blood}}}\int_{0}^{t}S_{\mathrm{blood}}\mathrm{dt}$$

The input signals - the drug as it moves from the digestive tract into the bloodstream, S_digest_, and the substance produced by the process, S_process_ - are added and integrated, yielding the output of the block, the blood drug level S_blood_ . To account for the body’s metabolism, a fraction 1/T_blood_ of the output signal is subtracted from the input.

### A3. The adaptive regulator

The input signals of the adaptive regulator are the drug administration and the sensor signal, processed by the loop control block. The sensor signal provides the information about the drug effect. The output of the adaptive regulator counteracts the disturbance by lowering the process output during the drug’s presence.

The adaptive regulator comprises a fast and a slow regulator. The fast regulator consists of the blocks “drug regulator”, “interval regulator” and “model estimation”. The slow regulator suppresses the slow changes in the input signal, its output being the average of the input signal. As the fast regulator reacts to fast changes only, the output of the slow regulator is subtracted from its input. It is assumed that the body more or less separately develops tolerance to the drug’s presence and to the intervals between drug administrations. The fast regulator therefore consist of a regulator which provides the adaptation to the drug’s direct effect and a regulator which provides adaptation to the interval between drug taking. The output of the complete adaptive regulator is a combination of signals from its individual components.

The model assumes the body to anticipate the effect of a drug to which it has developed tolerance. This implies that the body has made an estimate of what is going to happen when the drug is administered: it has a model of it. The organism also has made an estimate of the magnitude of the drug effect at the given state of tolerance development. These two entities are the main factors determining the functioning of the fast regulator: the level of tolerance development and the course of the drug effect.

### A3a. The fast regulator

The fast regulator consists of the blocks drug regulator,interval regulatorand model estimation(Figure A2). The input signal of the drug regulator S_d_ is multiplied by M_drug_, which represents the course of the drug level in the input signal over time. This signal is integrated (1/s) with a time constant T_drug_, yielding its average. The resulting value is a slowly rising signal, L_drug_. Multiplying L_drug_ by M_drug_ yields the output signal S_drug_.

Because of the slow response of the circuit, changes in the input magnitude will be followed only slowly by the output. The speed of change of the output magnitude - representing the slow development of tolerance by the organism - depends on the frequency of occurrence of the drug signal and the amplification of the feedback loop: 1/T_drug_. The relation between the signals is:

(3) $$S_{\mathrm{drug}}=M_{\mathrm{drug}}\cdot \frac{1}{T_{\mathrm{drug}}}\int_{0}^{t}(S_{d}-S_{\mathrm{drug}})\cdot M_{\mathrm{drug}}\mathrm{dt}$$

 and

(4) $$S_{\mathrm{drug}}=L_{\mathrm{drug}}\cdot M_{\mathrm{drug}}$$

The input to the interval regulator is obtained when the output signal of the drug regulator - S_drug_ - is subtracted from its top value L_drug_. The model of the interval is Mint.

The relation between the signals in the fast regulator describing the drug’s presence is then:

(5) $$S_{\mathrm{drug}}=M_{\mathrm{drug}}\cdot \frac{1}{T_{\mathrm{drug}}}\int_{0}^{t}(S_{d}-S_{\mathrm{drug}})\cdot M_{\mathrm{drug}}\mathrm{dt}-M_{\mathrm{drug}}\cdot \frac{1}{T_{\mathrm{decline}}}\int_{0}^{t}\frac{S_{\mathrm{drug}}}{M_{\mathrm{drug}}}\mathrm{dt}$$

 and

(6) $$S_{\mathrm{drug}}=L_{\mathrm{drug}}\cdot M_{\mathrm{drug}}$$

Similarly, the equation describing the interval regulator is:

(7) $$S_{\mathrm{int}}=M_{\mathrm{int}}\cdot \frac{1}{T_{\mathrm{int}}}\int_{0}^{t}(L_{\mathrm{drug}}-S_{\mathrm{drug}}-S_{\mathrm{int}})\cdot M_{\mathrm{int}}\mathrm{dt}-M_{\mathrm{int}}\cdot \frac{1}{T_{\mathrm{decline}}}\int_{0}^{t}\frac{S_{\mathrm{int}}}{M_{\mathrm{int}}}\mathrm{dt}$$

 and

(8) $$S_{\mathrm{int}}=L_{\mathrm{int}}\cdot M_{\mathrm{int}}$$

The output of the interval regulator is S_int_ . The output signal of the total fast regulator is obtained by subtracting the interval signal from the top level of the drug signal:

(9) $$S_{\mathrm{out}}=L_{\mathrm{drug}}-S_{\mathrm{int}}$$

Figure A3 shows the implementation of the fast regulator in the mathematical simulation program Simulink (see Peper 2004b).

### A3b. Estimation of the drug effect in the adaptive regulator

As the duration of the drug administration is relatively short in most cases, it may be represented by a short pulse. The model of the course of the drug concentration when it enters the bloodstream - M_drug_ - is then computed by calculating the effect of a pulse with a magnitude of 1 on the digestive tract’s transfer function. The input of the interval is acquired when the signal “drug” is subtracted from its top value of 1. Multiplying this signal with the transfer of the digestive tract yields the model of the interval M_int_:

(10) $$M_{\mathrm{drug}}=\int_{0}^{t}\mathrm{dru}g_{\;}\mathrm{dt}-\frac{1}{T_{\mathrm{digest}}}\int_{0}^{t}M_{\mathrm{drug}}\mathrm{dt}$$

 and

(11) $$M_{\mathrm{int}}=\int_{0}^{t}(1-\mathrm{drug})\mathrm{dt}-\frac{1}{T_{\mathrm{digest}}}\int_{0}^{t}M_{\mathrm{int}}\mathrm{dt}$$

T_digest_ is the time constant of the digestive system.

### A3c. The slow regulator

The slow regulator models the long term adaptation to the drug effect. In the tolerant state, the slow adaptation causes the magnitude of the negative reaction after the drug effect to depend on the interval between drug administrations: an infrequently taken drug has a small effect during the interval, while a frequently taken drug causes a large rebound. The slow regulator counteracts the disturbance by lowering the level of the process by the average of the drug effect. Its input signal - the sensor signal, processed by the loop control block - provides the information about the drug effect. The average of the input signal is obtained by a low pass filter with a time constant T_slow_:

(12) $$S_{\mathrm{slow}}=\int_{0}^{t}S_{\mathrm{contr}}\mathrm{dt}-\frac{1}{T_{\mathrm{slow}}}\int_{0}^{t}S_{\mathrm{slow}}\mathrm{dt}$$

### A4. The process

The model does not incorporate the characteristics of the process and the process regulator. In a specific model of drug tolerance where the process is included, the effect of the process transfer on loop stability has to be controlled by the the loop control block.

### A5. Loop control

A loop control is an essential element in any regulated system. It incorporates the open loop amplification which determines the accuracy of the regulation and it provides the necessary conditions for stable operation of the negative feedback system. For stable operation, the regulation loop has to contain compensation for the effect of superfluous time constants: their effect on the signals in the loop has to be counteracted by circuits with an inverse effect. In the present form of the model, only the effect of the bloodstream on the regulation loop is counteracted as the transfer of the process and its regulator and the transfer function of the sensor are set at unity.

The relation between the input and the output of the loop control is:

(13) $$S_{\mathrm{sens}}=\int_{0}^{t}S_{\mathrm{contr}}\mathrm{dt}-\frac{1}{T_{\mathrm{blood}}}\int_{0}^{t}S_{\mathrm{sens}}\mathrm{dt}$$

### A6. The sensor

The sensor transforms the chemical signal S_blood_ - the blood drug level - into the signal S_sense_ . In the present model, this transformation is assumed to be linear and is set at 1. In specific models of physiological processes, this complex mechanism can be described more accurately. Stable operation then requires that the effect of its transfer on loop stability is controlled by the the loop control block.
